# Penile metastasis of sigmoid colon carcinoma: a rare case report

**DOI:** 10.1186/s12894-015-0014-9

**Published:** 2015-03-17

**Authors:** Zhengbang Dong, Chao Qin, Qijie Zhang, Lei Zhang, Haijing Yang, Jingdong Zhang, Fei Wang

**Affiliations:** Department of Dermatology, Zhongda Hospital, Southeast University, Nanjing, Jiangsu 210009 China; Department of Urology, First Affiliated Hospital of Nanjing Medical University, Nanjing, Jiangsu 210029 China

**Keywords:** Carcinoma, Penile metastasis, Sigmoid colon

## Abstract

**Background:**

Metastasis to penis usually arises from genitourinary organs, but in rare cases, metastasis comes from the sigmoid colon. Furthermore, very few cases of penile metastasis of primary sigmoid colon carcinoma have been reported.

**Case presentation:**

We described a case of a 53-year-old man with penile metastasis of sigmoid colon carcinoma along with a review of the literature. Physical examination revealed two subcutaneous nodules on the glans penis. Biopsy of the nodules showed that penile metastasis of sigmoid colon carcinoma.

**Conclusion:**

Metastasis of sigmoid colon carcinoma to the penis is extremely rare, which presents an advanced form of sigmoid colon carcinoma, therefore survival is extremely shortened. Although treatment of penile metastasis is almost always palliative, it is important to recognize this unusual manifestation so that timely appropriate treatment can be initiated. Early recognition may enhance survival rate of these patients.

## Background

Colorectal cancer commonly metastasizes to regional lymph nodes, the liver, the lung, and the peritoneum, but rarely to the penis. The vast majority of secondary penile tumors originate from genitourinary organs, among which the prostate and the bladder are the most common sites of primary cancer. Less than 400 cases of penile metastases have been reported since 1870 when Eberth discovered the first case [1]. Penile metastasis from sigmoid colon carcinoma rarely occurs. Here is a case of secondary penile metastasis from sigmoid adenocarcinoma, followed by discussion of its possible metastatic mechanisms and clinical implications.

## Case presentation

**Figure 1:**
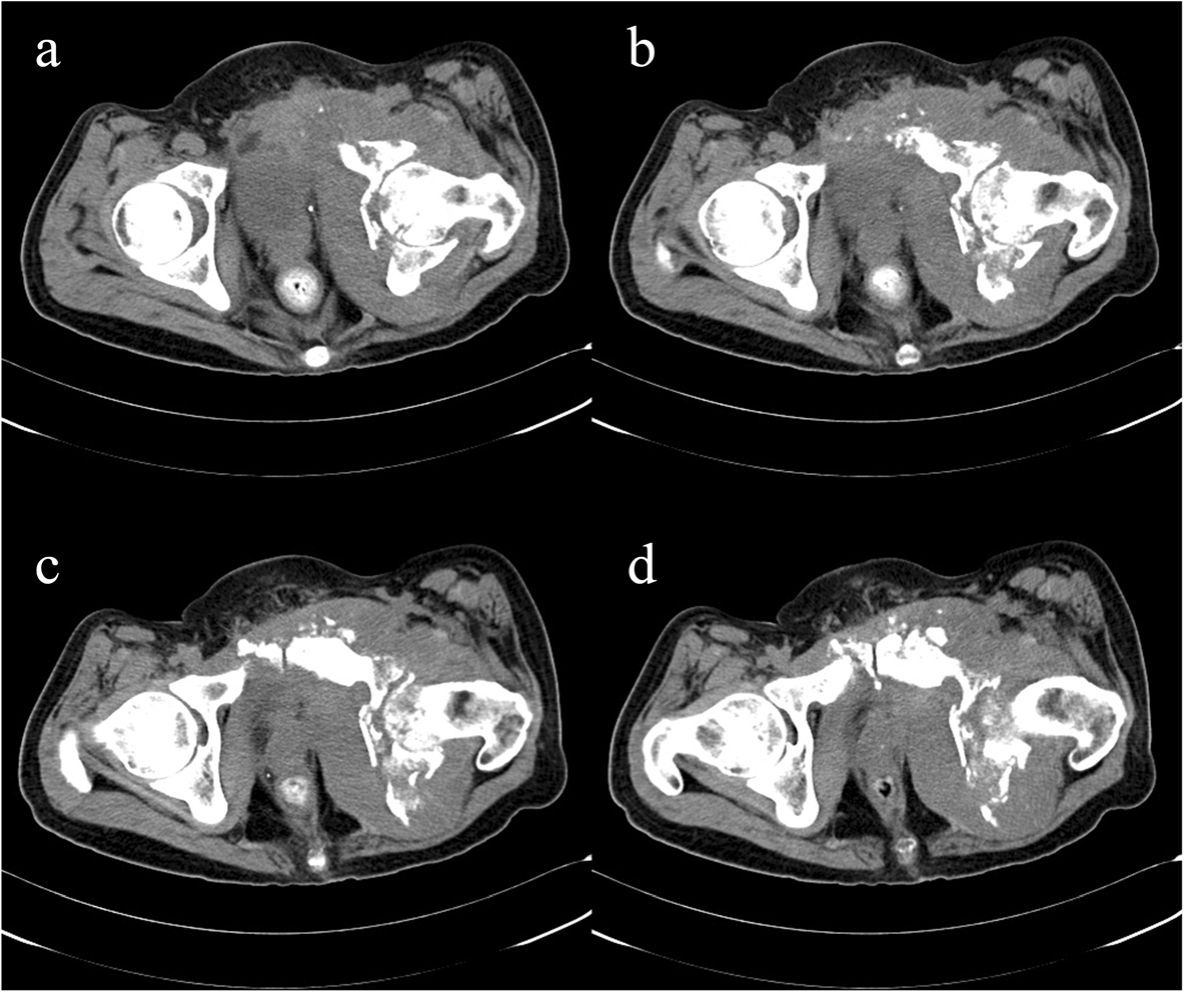
**CT indicates that the sigmoid colon carcinoma metastasizes to the bone and soft tissues. a**. The surrounding soft tissues of the left hip joint swell significantly and the spatium intermusculare disappears. **b**. The surrounding soft tissues of the left hip joint swell significantly and the spatium intermusculare disappears. **c**. Left acetabulum and pubic symphysis appear multiple bone destructions. **d**. Left acetabulum and pubic symphysis appear multiple bone destructions.

**Figure 2:**
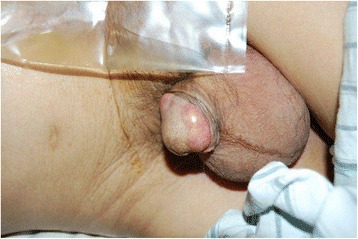
**Subcutaneous nodules on the glans penis.**

A 53-year-old Chinese man complaining of two painless lesions on the glans penis presented in December 2010. Five years ago, he had undergone a sigmoidectomy with a moderately well-differentiated adenocarcinoma. Postoperative histopathological examination revealed metastases in six regional lymph nodes (6 out of 13 nodes). After surgery he received adjuvant chemo-radiotherapy and regular follow-ups. In March 2008, serum car左髋臼cinoembryonic antigen level (CEA) markedly elevated (210.7 ng/ml). Computed tomographic (CT) [Figure 1] scan showed metastases to the lung and the pelvic cavity. Clinical examination revealed two painless nodules on his glans penis with angiotelectasis of itssurface. The nodules were pink, hard, and immobile, with a diameter of approximately 2 cm [Figure 2].

**Figure 3:**
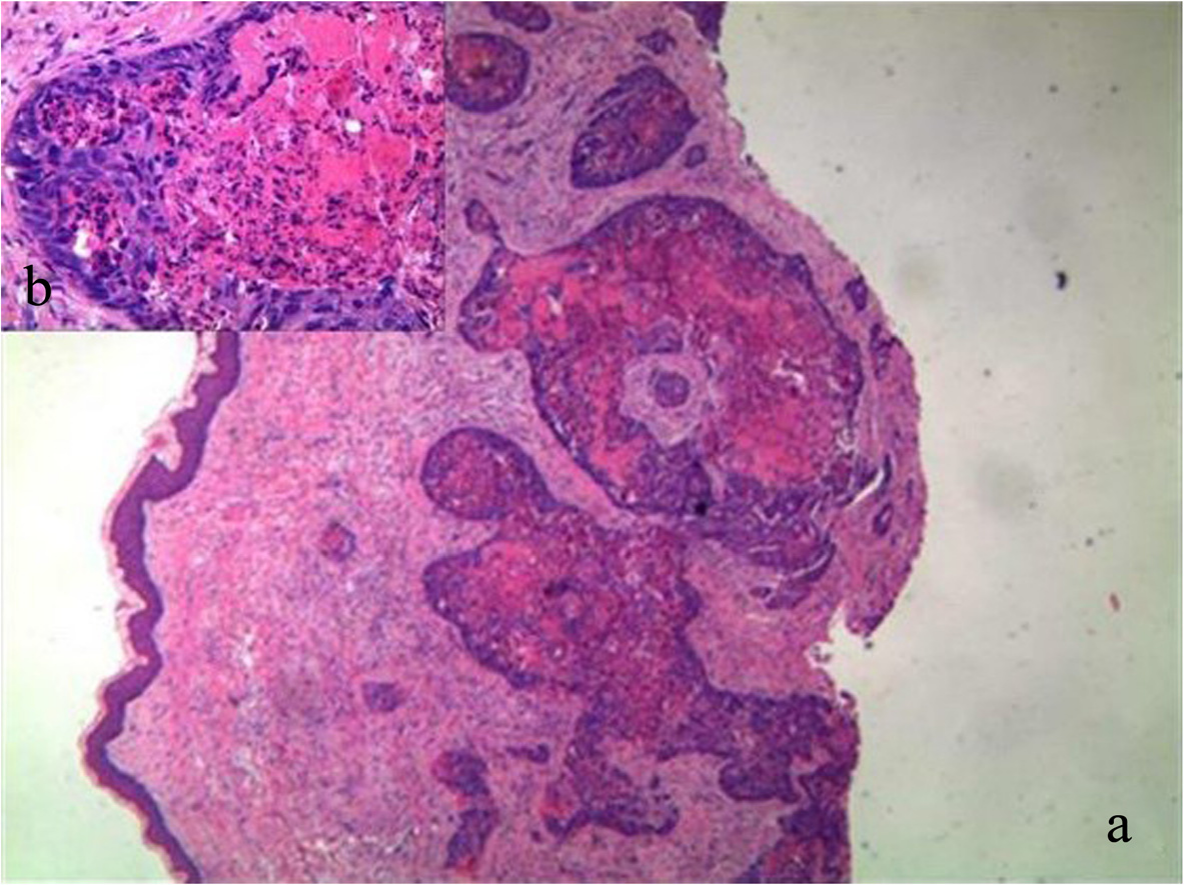
**Biopsy from a nodule of glans penis showing metastatic adenocarcinoma.** (H&E; **a** × 40, **b** × 400).

**Figure 4:**
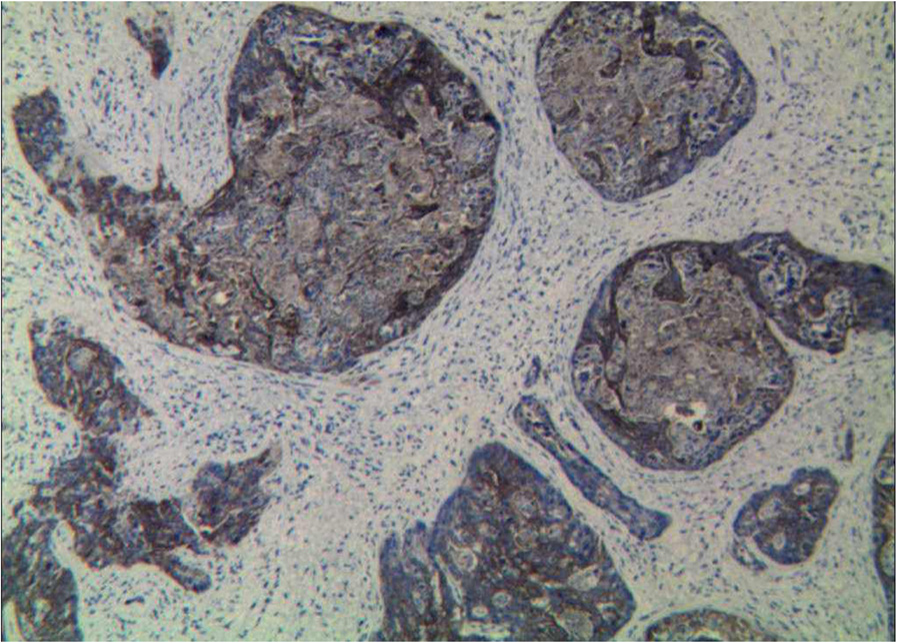
**Immunohistochemically the malignant cells were positive for cytokeratin 20.** (×100).

**Figure 5:**
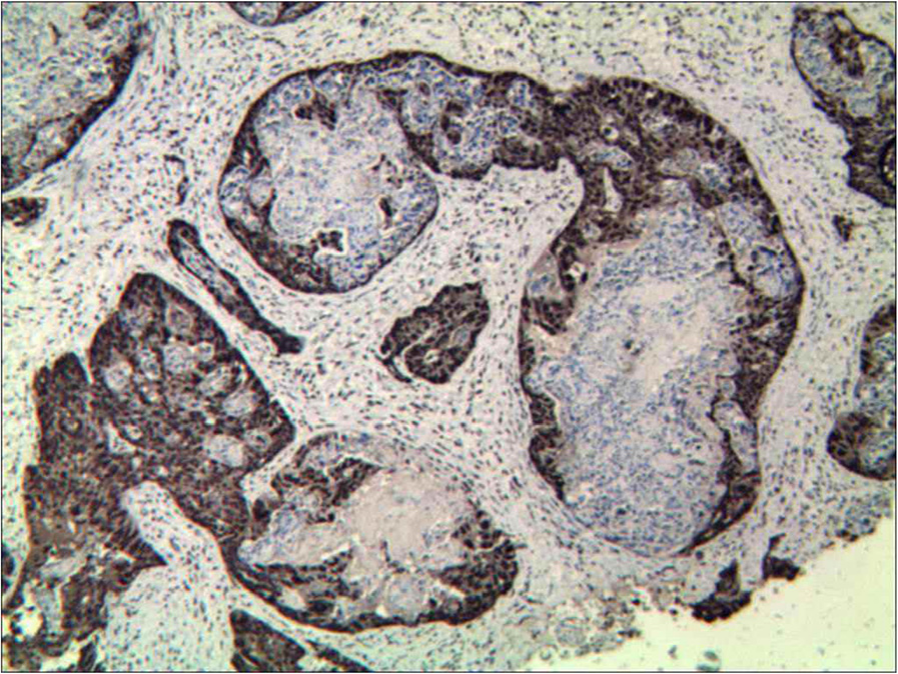
**Immunohistochemically the malignant cells were positive for CDX2.** (×100).

Senile malignancy was suspected, and a biopsy of the glans penis was performed. The biopsy of the left nodule led to a diagnosis of adenocarcinoma [Figure 3]. The cells exhibited pleomorphic, hyperchromatic nuclei with prominent nucleoli. Slides of the primary tumor were similar to those of the penile lesion,which was supported by immunohistochemistry in the metastatic deposit. The immunohistochemistry of tumor cells showed remarkable positive for cytokeratin 20 [Figure 4] and negative for cytokeratin 7. The cells were also positive for caudal-related homeobox transcription factor 2(CDX2) [Figure 5] and villin, while negative for cytokeratin 5/6 and cytokeratin 14. Therefore, it was concluded that the patient had penile metastasis from sigmoid colon carcinoma. Without further specific treatment, the patient just under symptomatic care survived for 2 months after being diagnosed with penile metastasis.

## Discussion

In spite of sufficient blood supply and close proximity to pelvic malignancies, penile metastasis rarely occurs, and the spread route is still in controversy. Cherian *et al*. explained as follows: (1) retrograde venous route, (2) retrograde lymphatic route, (3) direct extension, (4) implantation and secondary to instrumentation, (5) arterial spread [2]. More than one spread routes may occur in a single case, and it is very difficult to elucidate their exact mode of spread.

Metastasis of gastric carcinoma to the skin may manifest as cutaneous nodules, cellulitis, carcinoma en cuirasse, infiltrated skin plaques, carcinoma erysipelatoides, nodules, or large cauliflower-like papillomatous mass [3]. There is no characteristic symptom complex for secondary tumors of penis. The most common chief complaint was penile mass, followed by priapism. In up to 60% of the cases, the metastatic lesion presents as multiple infiltrative mass that is rigid, smooth, immobile, and painless. The penile shaft is the most affected anatomical site [1]. Priapism is a prominent feature in nearly 27% of patients. It could be caused either by tumor infiltration of cavernosal spaces, or by occlusion of draining veins due to infiltrating tumor cells. Irritation of neural pathways responsible for erection may be another mechanism [4]. Pain is not a prominent symptom in most of the patients. Other manifestations of penile metastasis include ulceration, obstructive or irritating voiding symptoms, hematuria, enlargement of inguinal lymph nodes, and penile or perineal pain [5,6].

The main differential diagnosis focuses on primary benign tumors, syphilitic chancre, venereal or other infectious ulcerations, idiopathic priapism, Peyronie’s plaque, candidiasis, cavernositis, tuberculosis of penis, sclerosing lipogranuloma [7]. Compared with ultrasound or computed tomographic scans alone, MRI (magnetic resonance imaging) is a favorable imaging modality to delineate the degree of involvement in the penis [8]. MRI is being increasingly used to state the disease; but accurate diagnosis of metastasis to penis must rely on penile lesion biopsy. In particular, immunohistochemistry is helpful in determining the primary site. Persistent painful penile nodule could raise suspicion of the existence of metastatic lesion. To achieve a diagnostic approach to these penile nodules, fine-needle aspiration (FNA) rather than complicated and invasive procedures can be performed, which can also be complemented by immunocytochemical phenotypical characterization to distinguish the primary site of origin [9].

About 90% of the reported penile metastases are part of widespread diseases. In the previously reported cases, the patients’ average survival is 9 months approximately, with a maximum of 18 months. The longest survival reported is 9 years [6]. Longer survival occurs in patients without other apparent metastatic sites. Clinical evidence of penile metastasis in a patient with a known malignancy is an ominous sign that should alert the clinicians of the dismal prognosis. The patient studied in this paper died of metastatic disease 2 months after penile metastases.

Given the poor prognosis, a stopgap measure to improve the patients’ life is usually taken into consideration. Treatment options include surgical excision, radiotherapy, and chemotherapy. The choice depends mainly on patients’ general clinical situation: type and extent of the primary tumor, presence of widespread metastatic disease, and type of symptoms [10]. Local surgical excision or radiotherapy is usually the most preferred. Penile ulcer has to be taken out by local excision. Radiotherapy is used to reduce the size of lesion as well as to control pain. Second-line chemotherapy with docetaxel, paclitaxel, vinorelbine, gemcitabine, irinotecan, and gefitinib has anindefinite curative effect [5]. Some patients only receive symptomatic treatment. A penile dorsal nerve block using local anesthesia may be of some benefit to control pain. Anxiety and pain can be dealt with parenteral opioids and anxiolytics. In the case with dismal prognosis, the patient has to be treated mainly with palliative therapy to relieve the intolerable symptoms.

## Conclusion

Metastasis of sigmoid colon carcinoma to the penis is extremely rare, which presents an advanced form of sigmoid colon carcinoma, therefore survival is extremely shortened. Although treatment of penile metastasis is almost always palliative, it is important to recognize this unusual manifestation so that timely appropriate treatment can be initiated. Early recognition may enhance survival rate of these patients.

## Consent statement

The patient has signed his written informed consent to participate in the study. The data do not contain any information that could identify the patient.
